# Nonadaptive host‐use specificity in tropical armored scale insects

**DOI:** 10.1002/ece3.6867

**Published:** 2020-11-04

**Authors:** Daniel A. Peterson, Nate B. Hardy, Geoffrey E. Morse, Takao Itioka, Jiufeng Wei, Benjamin B. Normark

**Affiliations:** ^1^ Department of Biology and Graduate Program in Organismic and Evolutionary Biology University of Massachusetts Amherst MA USA; ^2^ Department of Entomology and Plant Pathology Auburn University Auburn AL USA; ^3^ Department of Biology University of San Diego San Diego CA USA; ^4^ Graduate School of Human and Environmental Studies Kyoto University Kyoto Japan; ^5^ College of Agriculture Shanxi Agricultural University Taigu China

**Keywords:** Diaspididae, ecological specialization, herbivory, host range, niche breadth, polyphagy

## Abstract

Most herbivorous insects are diet specialists in spite of the apparent advantages of being a generalist. This conundrum might be explained by fitness trade‐offs on alternative host plants, yet the evidence of such trade‐offs has been elusive. Another hypothesis is that specialization is nonadaptive, evolving through neutral population‐genetic processes and within the bounds of historical constraints. Here, we report on a striking lack of evidence for the adaptiveness of specificity in tropical canopy communities of armored scale insects. We find evidence of pervasive diet specialization, and find that host use is phylogenetically conservative, but also find that more‐specialized species occur on fewer of their potential hosts than do less‐specialized species, and are no more abundant where they do occur. Of course local communities might not reflect regional diversity patterns. But based on our samples, comprising hundreds of species of hosts and armored scale insects at two widely separated sites, more‐specialized species do not appear to outperform more generalist species.

## Graphical abstract

In tropical canopies, we find evidence that diet specialization in armored scale insects might be nonadaptive. Diet generalists occur on more of their potential host plants and are no less abundant than diet specialists.

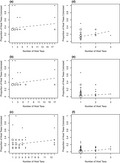

## 1 INTRODUCTION

High species diversity has been attributed to the partitioning of available resources into narrow ecological niches (Hutchinson, 1959). Yet, niche breadth varies greatly between species. Herbivorous insects are classic subjects for the study of this variation (Futuyma & Moreno, 1988; Hardy et al., 2020). Although diet specialists prevail, diet breadths vary continuously, and in some species are extremely broad (Forister et al., 2015; Normark & Johnson, 2011). How did this come to be?

For the most part, theorists have worked from the premise that diet specialization comes from genetic trade‐offs between adaptations to alternative resources, specifically antagonistic pleiotropy between alleles at a few diet‐determining loci (Futuyma & Moreno, 1988; Ravigné et al., 2009). Although empirical evidence for such genetic trade‐offs is scarce (Forister et al., 2012; Futuyma, 2008), they might be difficult to detect, as they can be hidden by interindividual fitness variation at linked loci (Joshi & Thompson, 1995), or arise from epistatic interactions between alleles (Celorio‐Mancera et al., 2016; Remold, 2012; Rodriguez‐Verdugo et al., 2014). In sum, the evidence is scarce for the adaptive trade‐off hypothesis, but it is difficult to falsify outright.

Alternatively, niche specialization could be driven by nonadaptive processes (Futuyma et al., 1995; Gompert et al., 2015). In fact, theoretical spatial models have shown that adaptive trade‐offs are not necessary to produce niche‐breadth distributions resembling those observed in natural communities (Forister & Jenkins, 2017), and we have evidence that in at least some species, populations are structured more by geographic variation than host‐use variation (Vidal et al., 2019). Evolving the ability to use a novel host almost certainly entails directional selection. But alleles promoting fitness on other potential hosts can be lost through genetic draft during strong directional selection on a novel host (Neher, 2013), or genetic drift when insect and host distributions cease to overlap (Gompert et al., 2015). One way or another, if host‐use traits are easy to lose but difficult to get back, neutral genetic processes could pull populations toward niche specialization (Hardy et al., 2016).

Host‐use trade‐offs in herbivorous insects have traditionally been investigated by comparing performance across multiple host plants of different insect genotypes within a population (e.g., Agosta et al., 2009). But a phylogenetically informed comparison of host use across multiple herbivore species offers a complementary perspective that may be less obscured by short‐term genetic contingencies (Funk et al., 1995; Futuyma, 2010; Hardy & Otto, 2014; Peterson et al., 2015, 2016). To wit, it could illuminate the overall relationship between diet breadth and ecological performance. If host‐use specificity is adaptive, we would expect that on any shared host specialists would tend to perform better than closely related generalists. Likewise, at the metapopulation level, if host‐use specificity is adaptive, we might expect specialists to do a better job of colonizing specific host resources (Gyllenberg & Metz, 2001). Conversely, if specificity is nonadaptive, we would expect generalists to colonize more of their potential hosts, and to perform just as well as specialists on shared hosts, or even perform better if there is a population‐genetic cost for specificity, for example, reduced population size and more erosive genetic drift.

We sought the evidence of such performance differences in the relative abundances and patch occupancies of 171 putative armored scale insect species (Hemiptera: Diaspididae) across 138 tree species in tropical rainforest communities on two continents. As is the case for herbivorous insects in general, most diaspidids are host‐use specialists, but some can be extremely polyphagous (García Morales et al., 2016). Diaspidids are sessile and have a simple, pathogen‐like life history in which new host trees are colonized by wind‐dispersed first‐instar nymphs that cannot survive for long away from a host (Hardy, 2018). A first‐instar nymph landing on a host thus experiences something like a no‐choice feeding trial, in which it either successfully develops, or dies. Once an individual starts to feed, it loses its legs and never moves to another feeding site. Potential for host choice is therefore limited, and the occurrence of later‐instar life stages on a plant is a clear indication that it is a suitable host for development and reproduction (Hill & Holmes, 2009). A female diaspidid completes her development, reproduces, and dies at the site where she settled as a first instar. A male regains motility as an adult—but adult males are completely nonfeeding and of course cannot establish new colonies on new host plants. Because dispersal is mediated by wind, it presumably cannot be adaptively directed toward favored hosts—rather, local plant species should be colonized in proportion to their abundance and apparency. The occurrence and abundance of a diaspidid species on a given host plant is a direct consequence of the ability of members of that diaspidid species to develop on that host. In other words, in diaspidids, there is an unusually simple and direct causal connection from ecological performance on a host to occupancy and abundance. Therefore, the occupancy and abundance of diaspidids on alternative hosts are useful indices of ecological performance on those hosts. Furthermore, with random, time‐limited dispersal, one might expect the greatest fitness for genotypes that perform best across most of the commonly encountered host plants. In fact, for diaspidids, we have previously shown that when host associations are treated as a binary use‐or‐nonuse traits, the phylogenetic patterns of host use are incompatible with strong adaptive trade‐offs (Peterson et al., 2015). Nevertheless, we have not previously been able to account for potentially important quantitative differences in performance across host‐plant groups.

Our approach was to (a) estimate allele genealogies among the sampled diaspidids for 3 loci, using DNA sequence data; (b) estimate species boundaries using these genealogies and also using morphology; (c) estimate the degree to which host use is phylogenetically conservative; (d) explicitly test for diet specialization in each species; and (e) use abundance‐based and patch‐occupancy‐based indices of performance to test whether more‐specialized species tend to do better than less‐specialized species on shared hosts.

## 2 METHODS

### 2.1 What to measure?

Diet breadth‐dependent performance trade‐offs could result from any number of mechanistic interactions between a herbivorous insect and a host plant. On a particular host plant, in comparison with a more‐specialized species, a relative generalist might have (a) a reduced ability to initiate feeding, (b) a lower feeding rate, (c) less efficient utilization of host nutrients, (d) greater susceptibility to host defenses, or (e) more exposure to natural enemies. No matter the mechanism, any trade‐offs that drive the evolution of specialization would need to ultimately limit survival or fecundity. If specialization is an adaptive response to trade‐offs between performance on alternative hosts, more‐specialized species should have higher survival or fecundity than less‐specialized species on shared resources. In the studied tropical forest plots, we were not able to measure survival or fecundity directly, but we were able to measure the abundance and patch occupancy of each diaspidid species on each host‐plant species. As mentioned earlier, because after the first‐instar stage each diaspidid is stuck for life on one host, an observation of a second instar or adult individual on a host is evidence of successful development on that host (Hill & Holmes, 2009). Moreover, the relative abundance of diaspidid species on each host‐plant species is an integrative proxy for fitness—integrating across host‐dependent differences in diaspidid fecundity and survival.

### 2.2 Sampling

We surveyed diaspidids at two wet lowland evergreen tropical rainforest sites: (a) San Lorenzo National Park, Panama, and (b) Lambir Hills National Park, Malaysia (on the island of Borneo). The Panama site, with 3,152 mm of rainfall per year, is at 30 m elevation near the Caribbean coast within a large expanse of protected forest. The Malaysia site, with 2,700 mm of rainfall a year, is at 160 m elevation in a small protected remnant forest that is now mostly surrounded by recently cleared areas converted to oil‐palm plantations. Both sites have high tree diversity; the Borneo site is particularly species‐rich, although that richness is dominated by the family Dipterocarpaceae (Basset et al., 2003). These sites were chosen because they provided access to the forest canopy with a crane, had tagged and data‐based each individual mature tree, and because the diaspidid faunas in those canopies were diverse. We were not able to search each tree in each plot, so we used the tree database at each site to divide tree specimens into sampling groups of one randomly selected individual per tree species. Only trees over 10 cm diameter at breast height were considered. Otherwise, our samples were not biased by the age or size of tree specimens (or the age and size of researchers). We did not sample any tree individual more than once, so tree species with only one individual were present only in the first round of sampling, those with two individuals were present in the first two rounds, and so on. This protocol allowed us to sample across the full diversity of host taxa while also getting multiple samples from common host species.

In Panama, we surveyed 90 trees over three rounds of sampling, representing 53 species, 48 genera, and 29 families (Table S1). In Malaysia, we surveyed 211 trees over 20 rounds of sampling, including 85 species, 48 genera, and 27 families (Table S2).

At each site, the canopy crane was used to access canopy foliage. From a gondola suspended from the canopy crane, at each focal tree we spent 20 person‐minutes searching accessible foliage. Any leaves and twigs that we saw were infested by scale insects, we cut from the tree and collected. From each tree, we also haphazardly took one 20‐cm twig sample and one 20‐cm^2^ bark sample. Removed plant material was stored in plastic bags and transferred to the laboratory for processing under magnification; live diaspidids were cut from the surrounding plant material and preserved in 95% ethanol. Specimens were subsequently sorted to life stage, and second instars and adult females were regarded as evidence of successful establishment.

### 2.3 Phylogenetics

DNA was extracted from all second‐instar and adult female armored scale insects using Qiagen DNeasy Blood & Tissue Kits (Qiagen) following the procedure outlined in Normark et al. (2019). We amplified three loci that have previously been used for diaspidid phylogenetics: elongation factor 1‐α (EF1α), part of the large ribosomal subunit rDNA gene (28S), and a part of the mitochondrial genome spanning cytochrome c oxidase I and II (COI‐II). PCR primers and protocols followed Andersen et al. (2010) and Gwiazdowski et al. (2011). PCR products were visualized using 1.5% agarose gels with SYBR Safe (Invitrogen), and successful reactions were purified with Exo SAP‐IT enzymatic digestion (Affymetrix). Sanger sequencing of the PCR products was completed by Macrogen or Eton Biosciences. DNA sequences have been submitted to GenBank under accession numbers MT641780–MT642048 and MT676866–MT677529; some sequences have been previously published in connection with phylogenetic studies, and these are given in Tables S1 and S2.

For each site, phylogenetic relationships among all sampled individuals were estimated from the DNA sequence data. Sequences from each genetic locus were aligned using PASTA (Mirarab et al., 2014), and alignments were trimmed to include only sites with nongap sequence for at least 80% of specimens (Capella‐Gutiérrez et al., 2009). Genealogies were inferred using the GTR + CAT model in RAxML (Stamatakis, 2014). The three single‐locus alignments were then combined as one supermatrix, from which we also inferred a phylogeny with RAxML. For use in comparative analyses, we made a version of the phylogeny with just one tip per species, and scaled branch lengths to time using an autocorrelated model of among‐lineage rate variation, fit with penalized likelihood as implemented in treePL (Smith & O’Meara, 2012), and constraining the armored scale root to be 50–75 million years old (Vea & Grimaldi, 2016).

### 2.4 Species delimitation and identification

In an attempt to make our inferences robust to errors in species delimitation, we delimited species in two ways. First, we delimited putative species with a version of the genealogical concordance method (as in Gwiazdowski et al., 2011). All clades shared by at least two gene trees, and not contradicted by the third gene tree, were considered evolutionarily independent lineages. Species were defined provisionally as the most inclusive independent lineages containing at least three terminal branches and no more exclusive independent lineages. This method precludes delimitation of species represented by fewer than three specimens. To work around this problem, we calculated the minimum divergence between provisional species clades and used that value as a maximum threshold for within‐species divergence. Any specimens separated by more than this distance from all other specimens were also considered distinct species.

We also delimited and identified species according to standard morphological criteria to the extent that this was possible. Because second instars and adults were both included in this study, whereas standard keys and descriptions are based on adults only, direct morphological comparisons and identifications were not always possible. The analyses below were repeated for DNA‐based and morphology‐based species delimitations. We retained all specimens in both analyses, whether or not they were morphologically identifiable; for the few specimens that were not morphologically identifiable, in the morphology‐based analysis we defaulted to the DNA‐based species.

### 2.5 Statistical analysis

We characterized host‐use specialization by diaspidid species in two ways, each applied at three levels of host‐plant taxonomy (species, genus, and family). This allowed us to assess the sensitivity of our inferences to different units of host‐plant diversity, and to measure the degree to which host‐use constraints were hierarchical. First, we quantified diet specificity; we asked whether diaspidids used less diverse hosts than expected by chance. Concretely, for each diaspidid species, we quantified host‐taxon diversity using Simpson's Reciprocal Diversity Index (RDI), which is essentially evenness‐corrected host‐taxon richness. We compared empirical RDIs to those expected under a null model of random host use. We simulated 1,000 null data sets by randomly permuting the associations between diaspidid species and individual host trees; then, for each permutation, we again calculated the mean RDI for the hosts of each diaspidid. With this approach, a diaspidid species is specialized to the extent that its host RDI is lower than expected under the null model.

In a second view of host‐use specialization, we calculated the phylogenetic conservatism of host use across diaspidid species. In other words, we asked whether evolutionary history constrains host use. We used the R package (R Core Team, 2017) MCMCglmm (Hadfield & Nakagawa, 2010) to measure the phylogenetic signal of host use by estimating the proportion of variance in the binary use or nonuse of each host taxon that could be explained by the diaspidid phylogeny. Empirical values for phylogenetic signal were then compared to those calculated under a null model. Null data sets were produced by randomly swapping associations between diaspidid species and host taxa until the associations were thoroughly shuffled (the number of random swaps was 10 times the overall number of associations). This preserved the empirical distribution of diet breadths while randomizing specific associations. *p‐*values for the empirical phylogenetic signal values were calculated using a *Z* test against each parameter's null data set values (which were approximately normally distributed). We corrected for multiple comparisons by assigning statistical significance according to a false discovery rate (FDR; Benjamini & Hochberg, 1995) of 0.05. The FDR procedure was conducted separately for each host‐taxon level because these analyses were not independent, and must be interpreted as alternative configurations of the same data.

We investigated the strength of performance trade‐offs by calculating for each host tree taxon the correlation between diaspidid diet breadth (count of host taxa) and mean abundance. If performance trade‐offs are strong, on any given host taxon, we expect more generalist (less‐specialized) species to be less abundant than more‐specialized species. We also investigated the relationship between diet breadth and the proportion of host trees of a taxon colonized at each site, as patch occupancy may be a better indicator of fitness than local abundance in a metapopulation of discrete colonies (Gyllenberg & Metz, 2001). Using R, we fit generalized linear models. For local abundance, the response variable was the number of diaspidid individuals identified per host tree, assuming a Poisson distribution. For metapopulation colonization rate, the response variable was the probability that an individual tree within each host taxon would be colonized by a diaspidid species, assuming a binomial distribution and excluding host taxa with fewer than three trees surveyed. Both models only incorporated data for host‐taxon‐by‐diaspidid associations with at least one record. To assess statistical significance, we compared empirical coefficients to those estimated from 1,000 null data sets, produced by randomly permuting the empirical data.

## 3 RESULTS

### 3.1 DNA‐based species delimitations

In Panama, we found live diaspidids on 75 trees, yielding 380 female specimens (adults and second instars). At least two loci were successfully amplified for 184 specimens, belonging to 53 DNA‐delimited species (Figure S1.4; Table S1.1). Assignment to a morphologically defined species was possible for 180 specimens, representing 32 described and 12 undescribed species. Species assignments and trophic links are in Table S1. In Malaysia, we found live diaspidids on 102 trees, yielding 480 female specimens. At least two loci were successfully amplified for 266 specimens, belonging to 123 DNA‐delimited species (Figure S1.5; Table S1.1). Assignment to a morphologically defined species was possible for 259 specimens, representing 20 described and 58 undescribed species. Species assignments and trophic links are in Table S2.

We found strong evidence for host‐use specialization, in terms of both less‐than‐expected host‐plant diversity and more‐than‐expected phylogenetic conservatism of host use. Simpson's RDI of each diaspidid species’ diet was significantly lower than expected at all host‐taxonomic levels and in both locations, except at the host‐species level in Panama (Table 1). Phylogenetic signal was significantly stronger than its null expectation for 19 host taxa (Figure S1.6), although it was higher at the Malaysia site (mean 0.61) than the Panama site (mean 0.45), with 18 Malaysian host taxa with significant phylogenetic conservatism, compared to just one Panamanian host taxon with significant conservatism.

**TABLE 1:** Mean Simpson's reciprocal diversity index (1/D) of individual host trees colonized by each DNA‐delimited diaspidid species for both sampling locations and all three host‐taxonomic levels

Location	Taxon level	Empirical 1/*D*	Null 1/*D*	*Z*	*p*
Panama	Species	3.162	3.321	−1.449	.147
Panama	Genus	3.008	3.295	−2.721	.007
Panama	Family	2.671	2.983	−2.887	.004
Malaysia	Species	1.643	2.087	−9.902	<.001
Malaysia	Genus	1.461	1.955	−6.400	<.001
Malaysia	Family	1.461	1.785	−3.472	<.001

**FIGURE 1:**
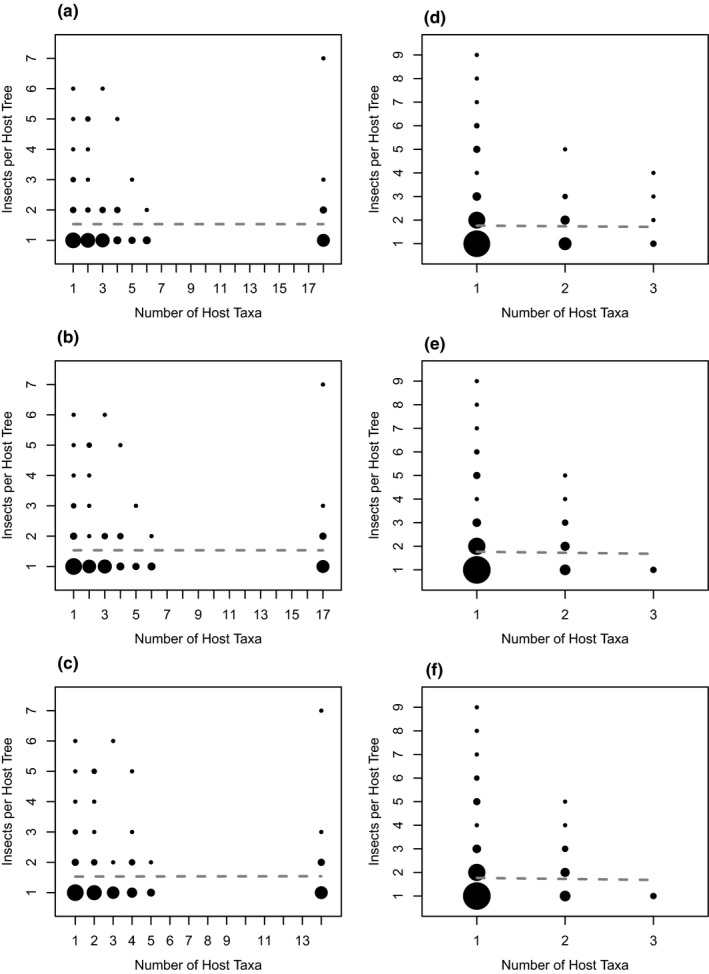
We found no relationship between the observed host range of diaspidid species and their abundance on each individual host. Here, we plot every tree colonized by each diaspidid species independently, and dot area is proportional to the number of data points at that coordinate. Results are divided by location and host‐taxonomic level: (a) Panama, species; (b) Panama, genus; (c) Panama, family; (d) Malaysia, species; (e) Malaysia, genus; and (f) Malaysia, family. None of these relationships (as fitted by a linear model, dashed line) was statistically different from expectations under a null model (all *p* > .9).

**FIGURE 2:**
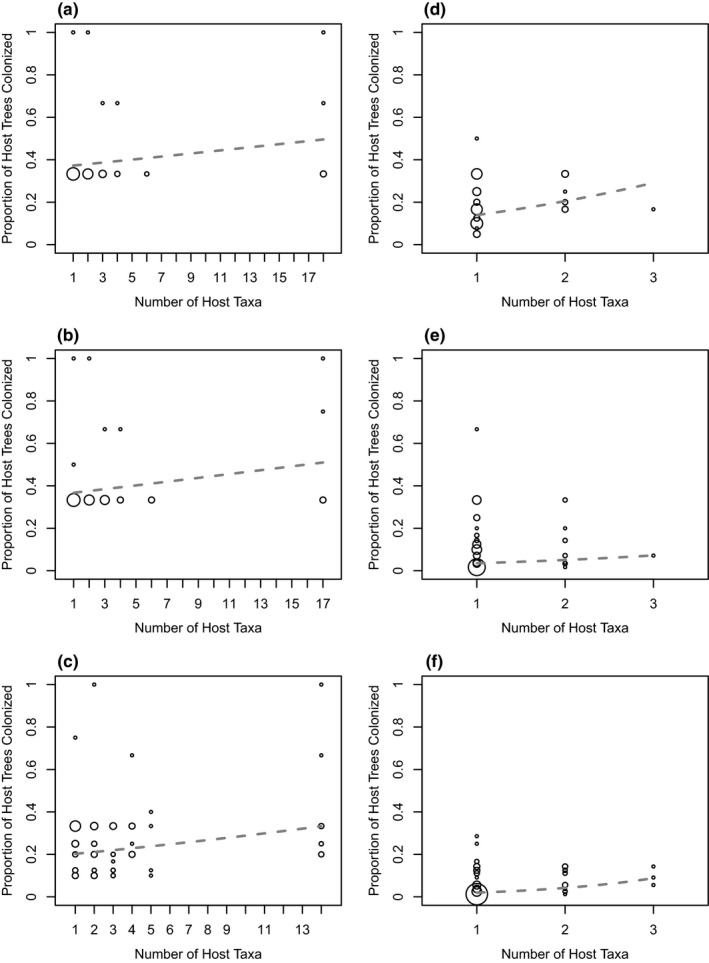
Diaspidid species with larger host ranges were present on a higher proportion of the individual trees in their host range. Here, each observed host‐taxon‐by‐diaspidid‐species interaction is plotted independently, although host taxa with fewer than three tree individuals surveyed were excluded from this analysis. Circle area is proportional to the number of data points at that coordinate. Results are divided by location and host‐taxonomic level: (a) Panama, species; (b) Panama, genus; (c) Panama, family; (d) Malaysia, species; (e) Malaysia, genus; and (f) Malaysia, family. All fitted slopes (dashed lines) were positive and all were statistically significant (*p* < .05), except in Malaysia by host genus (*p* = .062).

Despite the prevalence of diaspidid host‐use specialization at our two sites, and of extensive phylogenetic conservatism of host use in Malaysia, we found no evidence for performance trade‐offs on alternative hosts that would select against broad diets. More‐specialized species were no more abundant than less‐specialized species on specific host trees; the number of live adult or second‐instar female diaspidids found on each tree was not correlated with diet breadth (Figure 1; Table 2). Moreover, contra the metapopulation trade‐off hypothesis, Panamanian diaspidids with broader diets were observed on a higher proportion of the trees in their host taxa, although this effect was not significant for Malaysian diaspidids and their host‐plant species (Figure 2; Table 2).

**TABLE 2:** Statistical results from the models relating abundance per host and the proportion of host‐taxon occupancy to the local diet breadth of each DNA‐delimited diaspidid species

Location	Taxon level	Abundance			Occupancy proportion		
		Slope	*Z*	*p*	Slope	*Z*	*p*
Panama	Species	0.000	0.059	.953	0.030	2.616	.009
Panama	Genus	0.000	0.070	.944	0.036	3.125	.002
Panama	Family	0.001	0.074	.941	0.052	2.908	.004
Malaysia	Species	0.007	0.098	.922	0.361	2.077	.038
Malaysia	Genus	0.006	0.115	.909	0.455	1.867	.062
Malaysia	Family	0.006	0.111	.912	0.765	5.381	<.001

### 3.2 Morphology‐based delimitations

The results of the analyses using morphologically delimited species were broadly consistent with those using DNA‐delimited species. As for analyses using DNA‐delimited species, with morphology‐delimited species we found that diaspidid species were more‐specialized than expected by chance (Table S1.2), that more‐specialized species were no more abundant on their hosts (Table S1.3), and tended to occupy a smaller proportion of their potential host plants (Table S1.3). Actually, when modeling the links between Malaysian diaspidids and their hosts at the species level, we found positive effect of diet breadth on abundance that fell just short of significance (*p*‐value = .08).

## 4 DISCUSSION

Diaspidid species in tropical rainforest canopy habitats appear to use as hosts only a small proportion of the plant taxa in their local environment; simply put, as is the case for herbivorous insects in general (Forister et al., 2015), diaspidids tend to be exhibit diet specialization (Figure S1.7). But across the hundreds of trees that we surveyed, more‐specialized species were no more abundant on their hosts than more generalist species and occurred on a smaller proportion of their potential host plants. Is specialization for these diaspidids nonadaptive?

Such a conclusion would hinge on the assumption that what we saw within the reach of canopy cranes is what we would have seen elsewhere. But if abundance varies much over space, local differences in abundance could be misleading. Some such heterogeneity in the spatial distribution of diaspidids is expected. The quality of specific host‐plant resources can vary due to spatial mosaics of natural enemy pressure (Heard et al., 2006), as well as host‐plant features such as genotype, induced defensive state, and physical structure (Dixon, 2005). Although we saw no abundant species among those that were the most specialized, each could have been abundant somewhere else in the forest, where more suitable resources occur. Nevertheless, extreme patchiness in the abundance of the most specialized species would entail a metapopulation fitness cost, as local catastrophes would be more likely to cause extinction (Nurmi & Parvinen, 2008). In sum, potential spatial variation in abundance keeps us from making definite conclusions about the adaptiveness of specificity in diaspidids. But this potential is diminished by the cost of metapopulation patchiness and the consistency of our observations across species and communities.

Our inferences should be robust to temporal variation in the sampled diaspidid populations. Of course, when we sampled each site, some diaspidid species could have been under‐represented because of their phenology or the vagaries of local population dynamics. But we sampled many diaspidid species at each site, and see no basis to suspect that phenology would vary systematically with diet breadth, or that lows in stochastic populations fluctuations would occur predominantly in the most specialized species. In sum, species‐specific temporal variation in population size and age structure added statistical noise to our data, through which the signal of strong performance in generalists was strong enough to be discerned.

We found that the use of many host taxa by diaspidids was phylogenetically conservative. Although such conservatism of host use has been found for several other groups of herbivorous insects, such as butterflies (Janz et al., 2001) and beetles (Kelley & Farrell, 1998), it has a special significance for diaspidids, as they colonize new hosts haphazardly via wind (Magsig‐Castillo et al., 2010), and our previous work (Peterson et al., 2015), along with the research presented here, suggests that constraints on host use may be nonadaptive. Consequently, the phylogenetic conservatism of host use in diaspidids may more likely denote historical constraints on contemporary niches than long‐term niche optimization. Given the evidence of a lack of performance trade‐offs for diaspidids between alternative hosts (Peterson et al., 2015), host‐use constraints would seem to persist in the face of what may be strong selection for broad diets.

Our results also shed light on the complexity of host‐use traits in herbivorous insects (Barrett & Heil, 2012; Forister et al., 2012). We found that specialization in armored scale insects occurs at all three of the host‐taxonomic levels that we considered (species, genus, and family), suggesting that the genomic architecture of host‐use traits is both complex and hierarchical. The use of multiple hosts is often associated with close phylogenetic relationships among those hosts (Gilbert & Webb, 2007; Krasnov et al., 2012), yet such results in flying insects may reflect host preference or ease of host recognition more than host performance (Bernays, 2001). Because diaspidids have little opportunity to choose a host, phylogenetic conservatism at multiple taxonomic levels implies that performance on a host likely depends on many traits of various effect sizes. Although actual mechanisms are as yet unclear (but see Ali & Agrawal, 2012; Hogenhout & Bos, 2011), the involvement of many genetic loci in plant–insect interactions is consistent with both ecological (Singer & Stireman, 2005) and genetic (Remold, 2012) theory and recent genome‐wide association studies (e.g., Egan et al., 2015; Gompert et al., 2015).

Our DNA‐based species delimitations allow us some insight into whether any species that have been characterized as extremely polyphagous (Normark & Johnson, 2011; Normark et al., 2014) are in fact clusters of cryptic species that are more‐specialized. The answer is mixed. On the one hand, in Panama, the single most polyphagous species in the sample, *Selenaspidus articulatus* (Morgan), shows no hint of cryptic species diversity—not surprisingly, as it is native to Africa and invasive in Panama (Normark et al., 2019). On the other hand, several other reportedly highly polyphagous species do appear to represent cryptic species clusters. In Panama, only a single morphologically delimited species shows evidence of cryptic diversity: Samples of *Diaspis boisduvalii* (Signoret) were apportioned across five DNA‐delimited species. In contrast, at the Malaysian site, cryptic diversity appears rampant, especially among the most polyphagous species: *Chrysomphalus dictyospermi* (Morgan), purported to use 80 host families worldwide, was recovered as two cryptic species; *Chrysomphalus pinnulifer* (Maskell), with 40 host families worldwide, was also recovered as two cryptic species; *Morganella longispina* (Morgan), with 22 host families worldwide, was also recovered as three cryptic species; and *Aonidiella inornata* McKenzie, with 24 host families worldwide, was also recovered as three cryptic species. We also found cryptic diversity in less polyphagous Southeast Asian species: *Silvestraspis uberifera* (Lindinger), three cryptic species, and *Aulacaspis calcarata* (Takagi), eight cryptic species, and several undescribed species. Most strikingly, one undescribed species provisionally designated *Sishanaspis* ud4977 appears to comprise a complex of 10 cryptic species. The upshot is that in Malaysia traditional morphology‐based species delimitation seems to miss much of the true diversity. But our inferences about the extent and consequence of diet specificity in diaspidids appear robust to how species are delimited.

Although it falls outside of the main theme of this study, one other insight afforded by the morphological species identifications, which may help explain difference in diet breadth and host occupancy observed between the two sites, is the incidence of invasive species. At the Malaysian site, we found no genera native to regions other than Southeast Asia, whereas in Panama nearly half of morphologically identifiable individuals (77/180 = 43%) belong to invasive species (Normark et al., 2019). In addition to *Selenaspidus articulatus* (sampled on 18 host species), these include several genetically uniform populations that we sampled on multiple host species, including *Lepidosaphes rubrovittata* (Cockerell) (six hosts), *Chrysomphalus dictyospermi* (four hosts), *Aspidiotus excisus* Green (three hosts), and *Lepidosaphes punicae* Laing (three hosts). Thus, the narrower diets and higher host occupancy in Panama could have something to do with the relatively recent arrival of much of the diaspidid fauna, although such an effect of geographic range expansion on diet breadth would be the opposite of what has been found for some other herbivorous insects (Lancaster, 2020).

In conclusion, evolutionary fitness is difficult to measure and we cannot draw straight lines connecting it to differences in local abundance and patch occupancy. It could be that for diaspidids the quality of host resources is extremely uneven across tropical canopies and that for each of the relatively specialized species we sampled there was an unsampled population booming somewhere else in the forest. Or it could simply be that host specialization is not adaptive for wind‐dispersed plant pathogens in diverse host‐plant communities. If host‐use specialization is adaptive and high‐quality hosts are patchy and rare, then the question becomes this: Why are the most specialized species so much less abundant than expected? What are the conditions that must be met for a specialist to make good on their specialty? But as it stands, the patchiness and rarity of specialist‐supporting host plants are ad hoc hypotheses, that is, extraneous additions to the theory of adaptive host‐use specialization to prevent its falsification.

## CONFLICT OF INTEREST

The authors are unaware of any conflicts of interest.

## AUTHOR CONTRIBUTION

**Daniel A Peterson:** Conceptualization (equal); Data curation (equal); Formal analysis (equal); Investigation (equal); Methodology (equal); Visualization (equal); Writing‐original draft (equal); Writing‐review & editing (equal). **Nate B Hardy:** Data curation (equal); Formal analysis (equal); Investigation (equal); Visualization (equal); Writing‐original draft (equal); Writing‐review & editing (equal). **Geoffrey E Morse:** Conceptualization (equal); Data curation (equal); Funding acquisition (equal); Investigation (equal); Supervision (equal); Writing‐original draft (equal); Writing‐review & editing (equal). **Takao Itioka:** Data curation (equal); Investigation (equal); Writing‐review & editing (equal). **Jiufeng Wei:** Data curation (equal); Investigation (equal). **Benjamin Normark:** Conceptualization (equal); Data curation (equal); Funding acquisition (equal); Investigation (equal); Project administration (equal); Writing‐original draft (equal); Writing‐review & editing (equal).

## Supporting information

Figure S1‐1

Figure S1‐2

Figure S1‐3

Figure S1‐4

Table S1

Table S2

Supinfo

## Data Availability

DNA sequences are deposited in GenBank, and trophic link data and analysis scripts have been uploaded to Dryad (https://doi.org/10.5061/dryad.1vhhmgqr4).
