# Supplementary figures and images for: Nonadaptive host‐use specificity in tropical armored scale insects
Source: Ecol Evol. 2020 Nov 4;10(23):12910–9. doi: 10.1002/ece3.6867 (PMC7713922; doi:10.1002/ece3.6867)

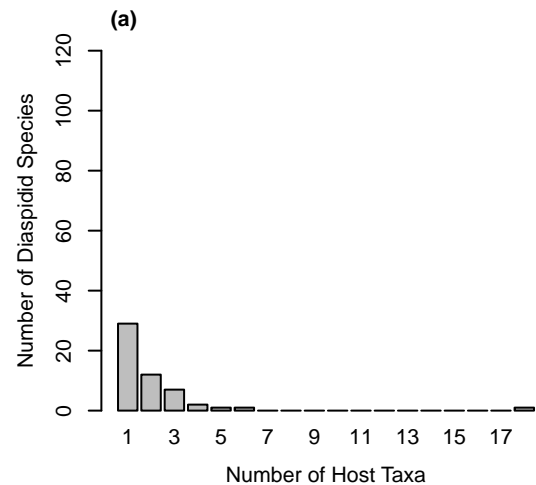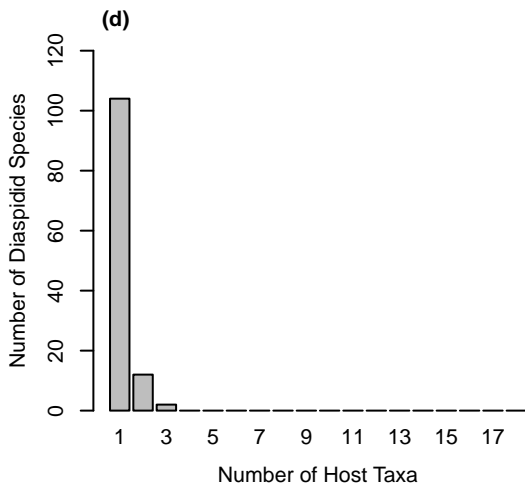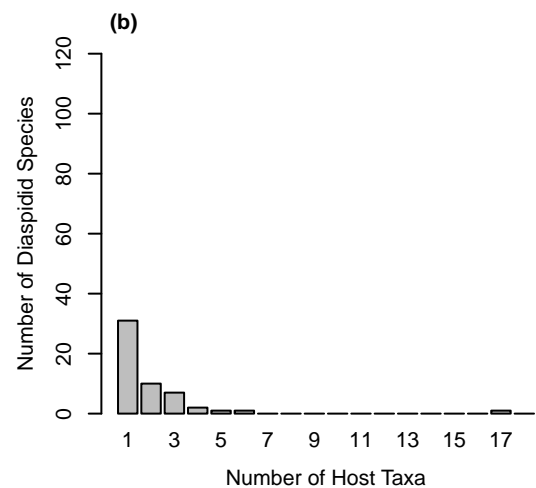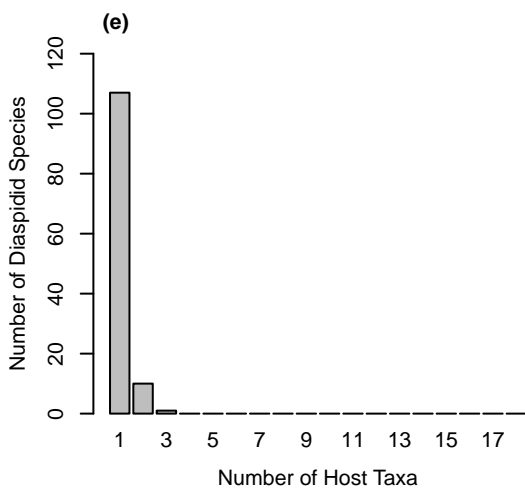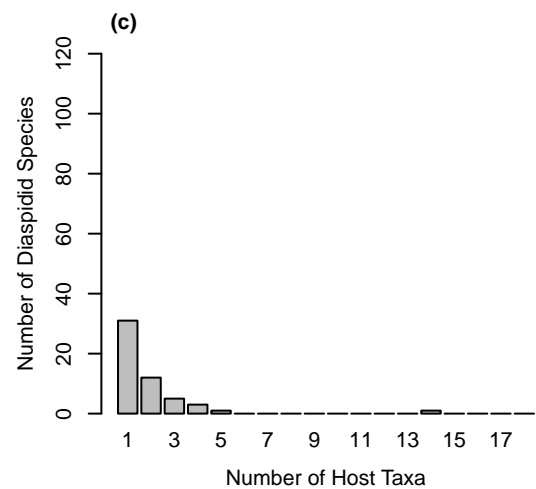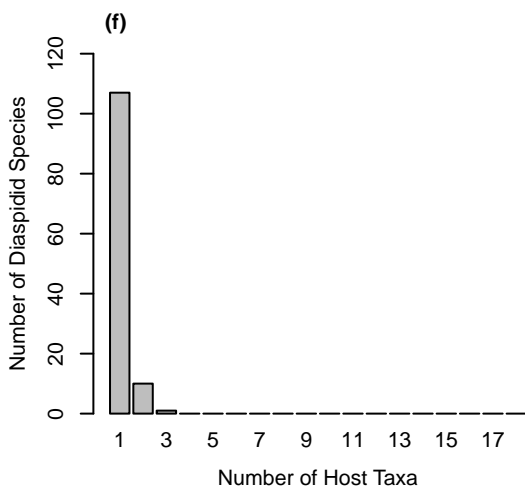

Supplement: Supplementary file 1 — Figure S1‐1 [file ECE3-10-12910-s001.pdf]

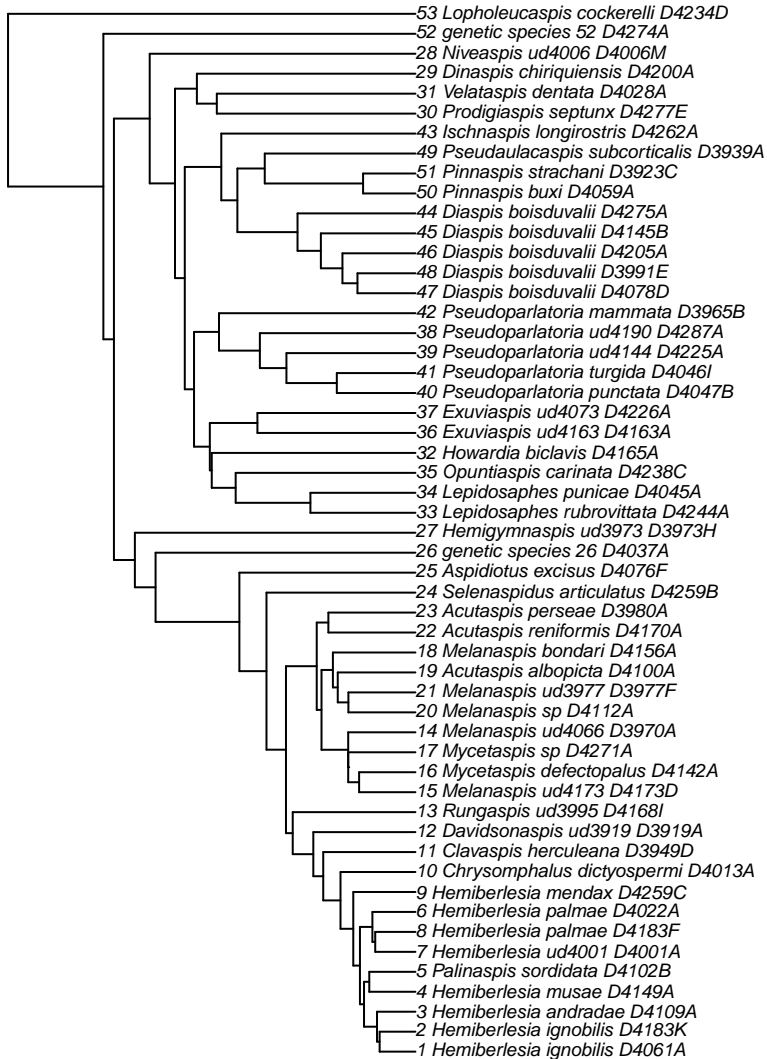

Supplement: Supplementary file 2 — Figure S1‐2 [file ECE3-10-12910-s002.pdf]

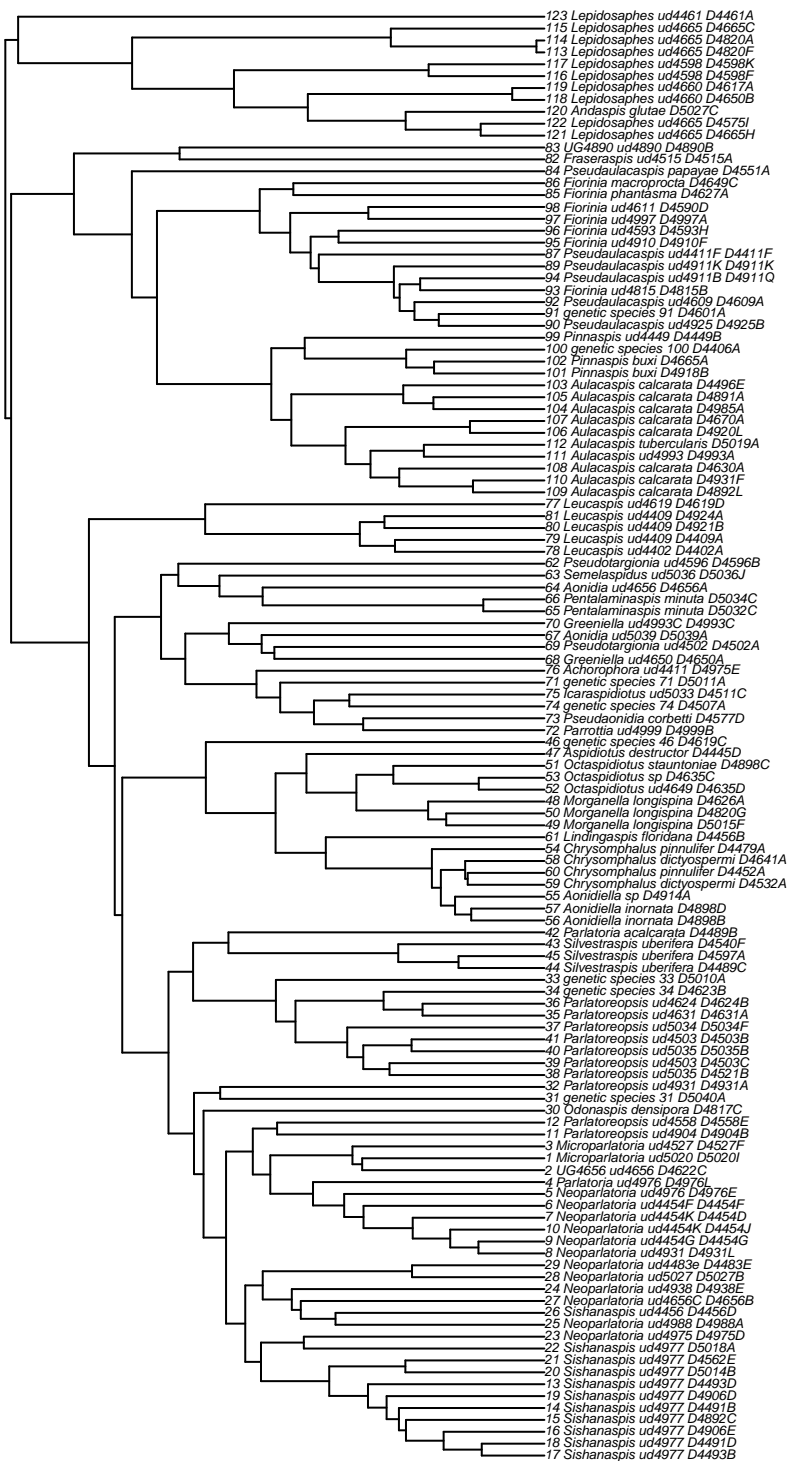

Supplement: Supplementary file 3 — Figure S1‐3 [file ECE3-10-12910-s003.pdf]

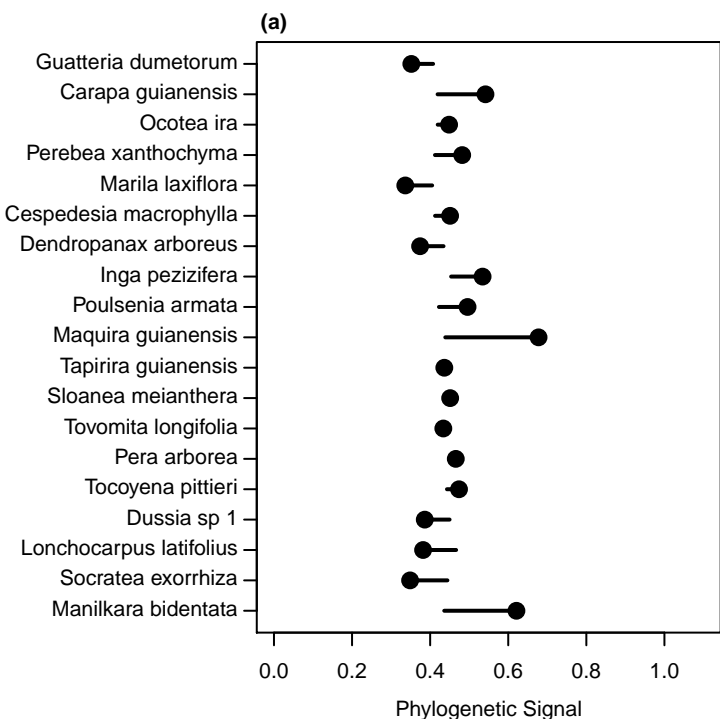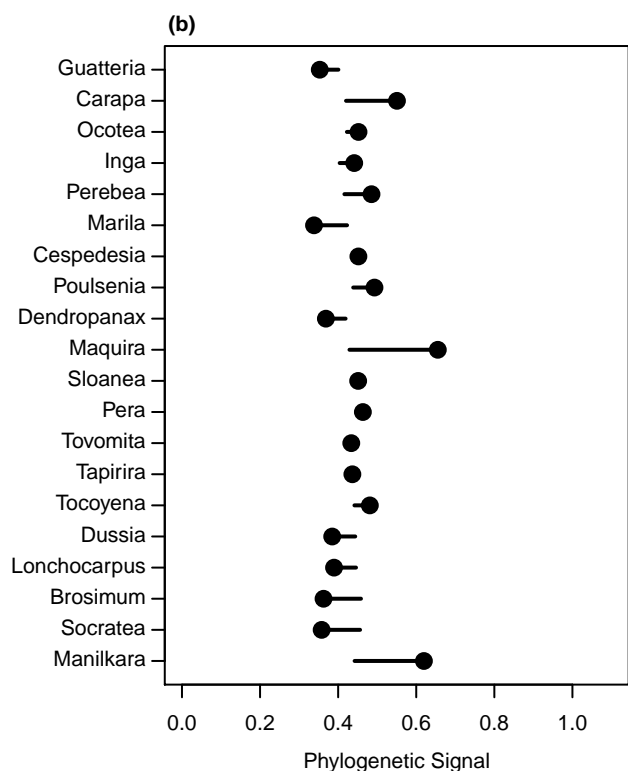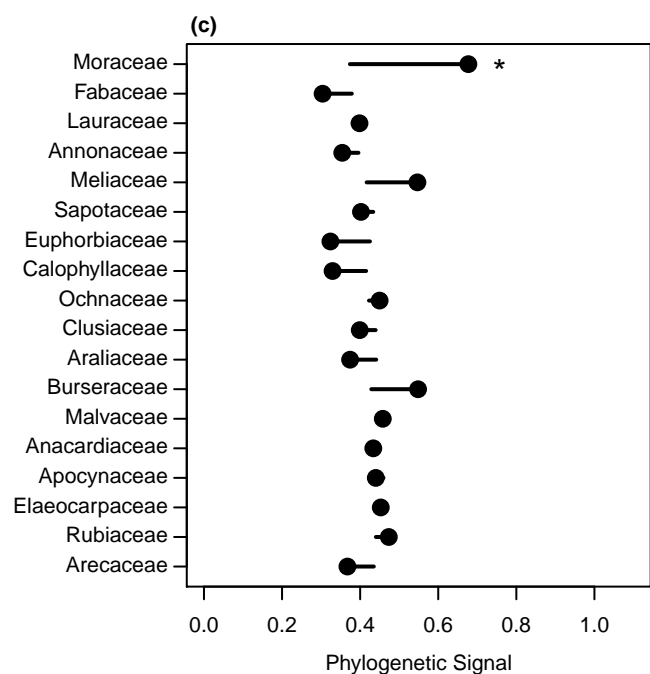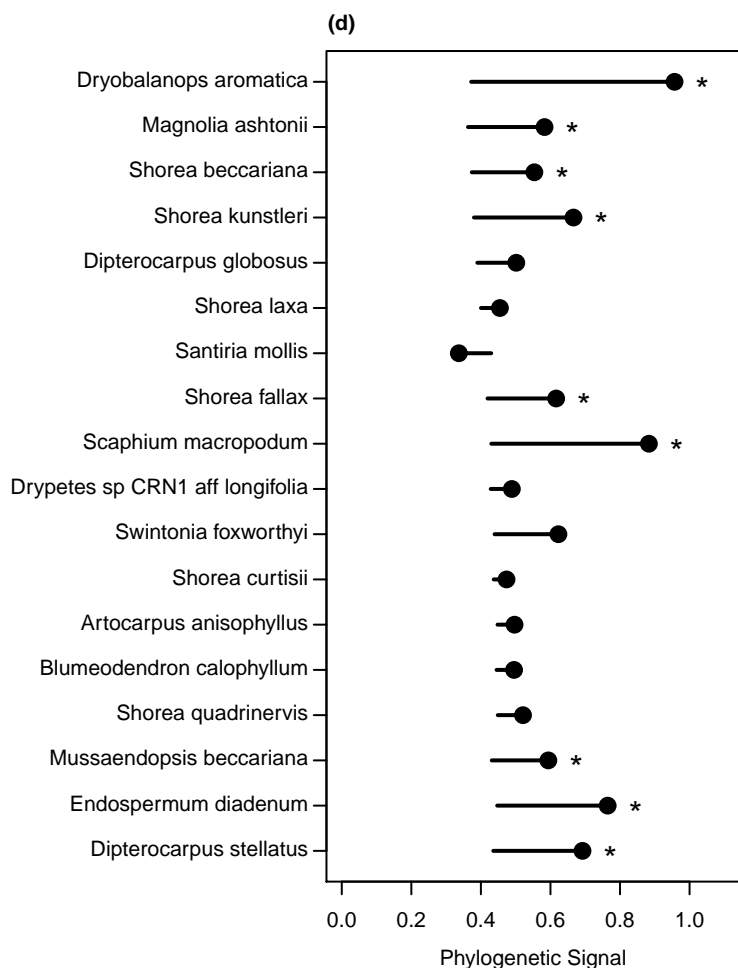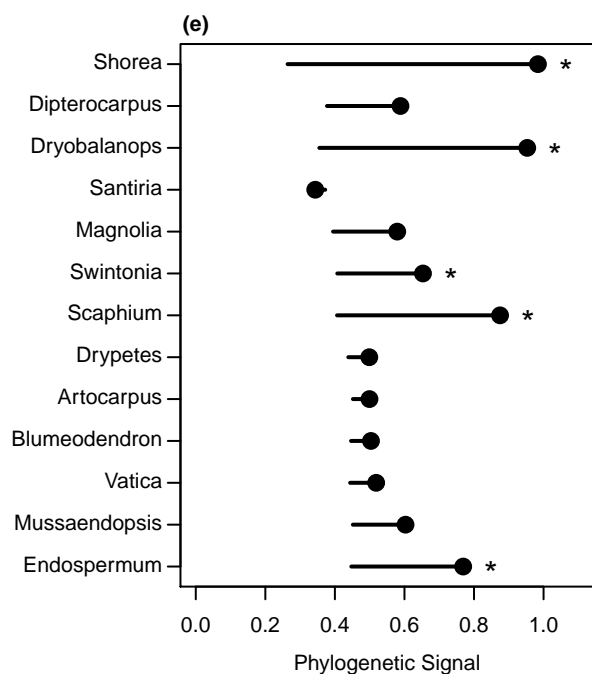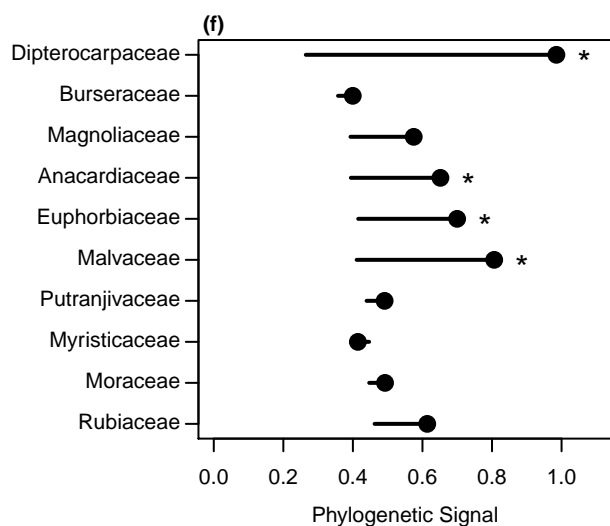

Supplement: Supplementary file 4 — Figure S1‐4 [file ECE3-10-12910-s004.pdf]
