# Supplementary material for: Nonadaptive host‐use specificity in tropical armored scale insects
Source: Ecol Evol. 2020 Nov 4;10(23):12910–9. doi: 10.1002/ece3.6867 (PMC7713922; doi:10.1002/ece3.6867)
Supplement: Supplementary file 7 — Supinfo [file ECE3-10-12910-s007.docx]

**Peterson, Hardy et al. Appendix 1**

**Table A1.1:** Trimmed genetic sequence alignment data for diaspidid specimens collected in Panama and Malaysia.

| **Location** | **Locus** | **Number of  Specimens** | **Alignment  Length (bp)** | **Proportion  Missing** | **Proportion  Variable sites** | **Proportion  Parsimony Informative** |
| --- | --- | --- | --- | --- | --- | --- |
| Panama | 28S | 290 | 520 | 0.02 | 0.59 | 0.53 |
| Panama | COI_II | 212 | 745 | 0.00 | 0.74 | 0.68 |
| Panama | EF1a | 206 | 747 | 0.04 | 0.53 | 0.48 |
| Malaysia | 28S | 359 | 611 | 0.04 | 0.55 | 0.47 |
| Malaysia | COI_II | 271 | 734 | 0.02 | 0.86 | 0.80 |
| Malaysia | EF1a | 234 | 798 | 0.08 | 0.53 | 0.45 |

**Table A1.2:** Mean Simpson’s reciprocal diversity index (1/D) of individual host trees colonized by each morphology-delimited diaspidid species for both sampling locations and all three host taxonomic levels.

| **Location** | **Taxon Level** | **Empirical 1/D** | **Null 1/D** | ***Z*** | ***P*** |
| --- | --- | --- | --- | --- | --- |
| Panama | Species | 3.224 | 3.361 | -1.361 | 0.17 |
| Panama | Genus | 3.082 | 3.335 | -2.59 | 0.0096 |
| Panama | Family | 2.769 | 3.024 | -2.31 | 0.02 |
| Malaysia | Species | 1.874 | 3.015 | -4.575 | < 0.001 |
| Malaysia | Genus | 1.843 | 2.543 | -4.756 | < 0.001 |
| Malaysia | Family | 1.843 | 2.095 | -1.97 | 0.048 |

**Table A1.3:** Statistical results from the models relating diet breadth to abundance per host and proportion of host taxon occupancy for each morphology-delimited diaspidid species.

| **Location** | **Taxon Level** | **Abundance** | | | **Occupancy proportion** | | | |
| --- | --- | --- | --- | --- | --- | --- | --- | --- |
|  |  | **Slope** | ***Z*** | ***P*** | | **Slope** | ***Z*** | ***P*** |
| Panama | Species | 0.001 | 0.205 | 0.838 | | 0.028 | 2.517 | 0.012 |
| Panama | Genus | 0.002 | 0.167 | 0.868 | | 0.035 | 2.966 | 0.003 |
| Panama | Family | 0.003 | 0.288 | 0.773 | | 0.053 | 2.946 | 0.003 |
| Malaysia | Species | 0.058 | 1.736 | 0.083 | | 0.040 | 1.108 | 0.268 |
| Malaysia | Genus | 0.033 | 0.549 | 0.583 | | 0.355 | 2.537 | 0.011 |
| Malaysia | Family | 0.004 | 0.098 | 0.922 | | 0.721 | 5.905 | < 0.001 |


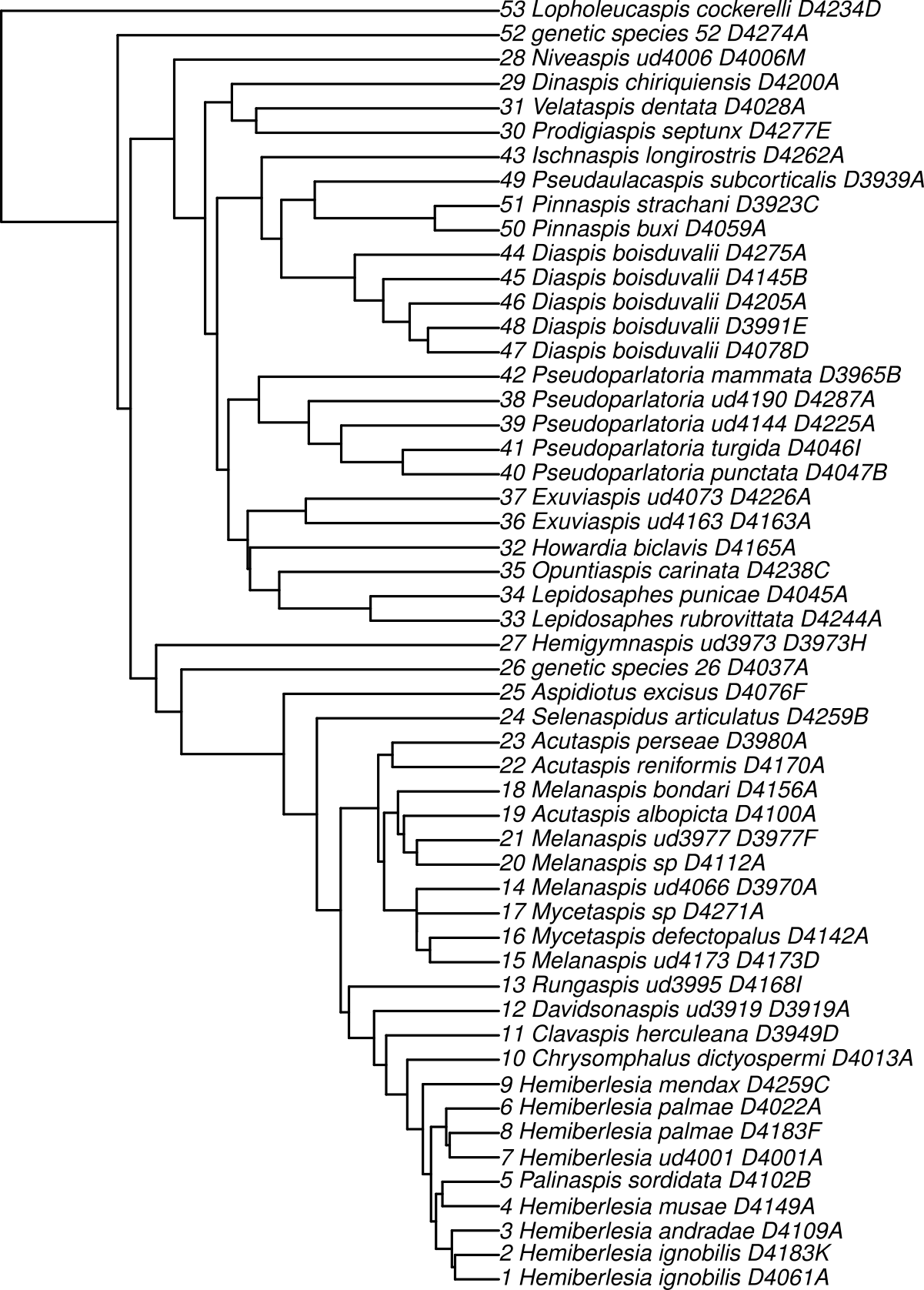


**Figure A1.4:** Phylogeny of diaspidid species sampled in Panama, estimated from three loci using maximum likelihood, and with branch lengths scaled to time with penalized likelihood. Each tip is labeled with first the genetic species assignment, then the morphological species assignment, and then an accession number. Only one exemplar is shown for each genetic species.


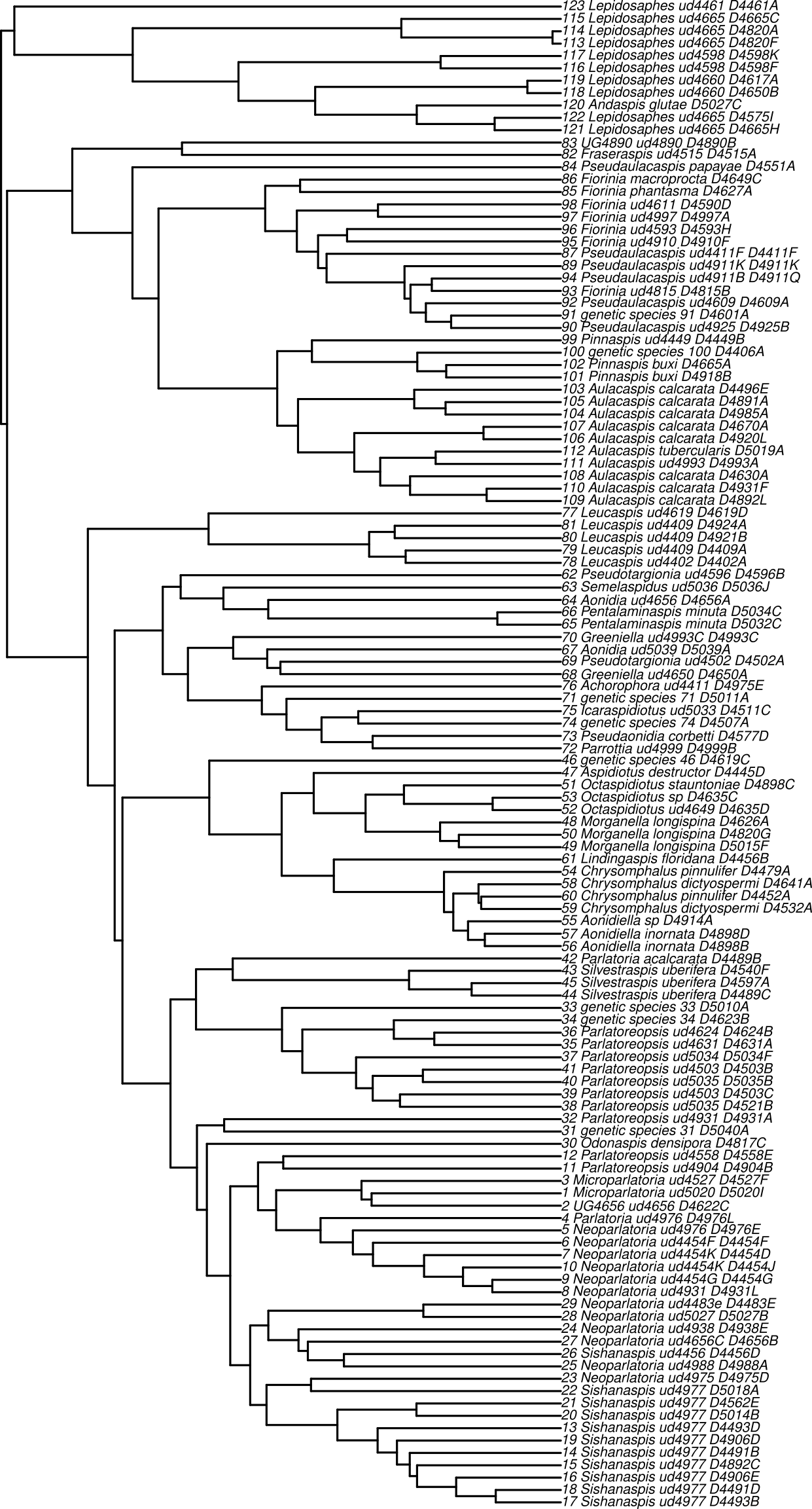


**Figure A1.5:** Phylogeny of morphologicaly-delimited diaspidid species sampled in Malaysia, estimated from three loci using maximum likelihood, and with branch lengths scaled to time with penalized likelihood. Each tip is labeled with first the genetic species assignment, then the morphological species assignment, and then an accession number. Only one exemplar is shown for each genetic species.


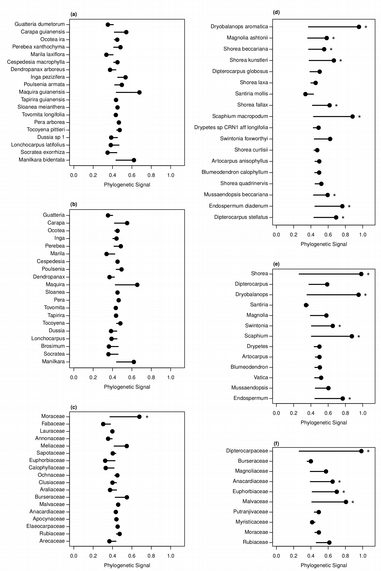


**Figure A1.6:** Phylogenetic signal of host-use. Here the dot indicates the empirical proportion of the host-use variation explained by the diaspidid phylogeny, and the line is drawn to the expected proportion under the null model of random host associations. Empirical values significantly different from the expected value (with a false discovery rate of 0.05) are marked with an asterisk. Results are divided by location and host taxonomic level: a) Panama, species; b) Panama, genus; c) Panama, family; d) Malaysia, species; e) Malaysia, genus; f) Malaysia, family.


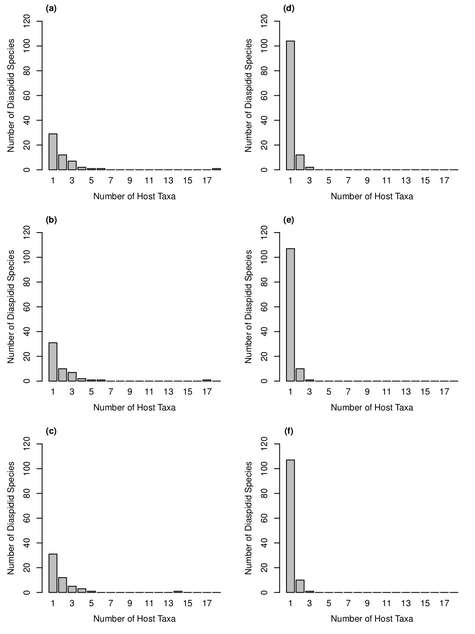


**Figure A1.7:** Histograms of the number of diaspidid species with each host-range size. Results are divided by location and host taxonomic level: a) Panama, species; b) Panama, genus; c) Panama, family; d) Malaysia, species; e) Malaysia, genus; f) Malaysia, family.
